# Tree height–diameter allometry across the United States

**DOI:** 10.1002/ece3.1328

**Published:** 2015-02-20

**Authors:** Catherine M Hulshof, Nathan G Swenson, Michael D Weiser

**Affiliations:** 1 Departamento de Biología, Recinto Universitario de Mayagüez, Universidad de Puerto RicoMayagüez, Puerto Rico, 00681; 2 Department of Plant Biology, Michigan State UniversityEast Lansing, Michigan, 48824; 3 Department of Biology, University of OklahomaNorman, Oklahoma, 73069

**Keywords:** Allometry, angiosperm, Forest Inventory and Analysis National Program, gymnosperm, scaling

## Abstract

The relationship between tree height and diameter is fundamental in determining community and ecosystem structure as well as estimates of biomass and carbon storage. Yet our understanding of how tree allometry relates to climate and whole organismal function is limited. We used the Forest Inventory and Analysis National Program database to determine height–diameter allometries of 2,976,937 individuals of 293 tree species across the United States. The shape of the allometric relationship was determined by comparing linear and nonlinear functional forms. Mixed-effects models were used to test for allometric differences due to climate and floristic (between angiosperms and gymnosperms) and functional groups (leaf habit and shade tolerance). Tree allometry significantly differed across the United States largely because of climate. Temperature, and to some extent precipitation, in part explained tree allometric variation. The magnitude of allometric variation due to climate, however, had a phylogenetic signal. Specifically, angiosperm allometry was more sensitive to differences in temperature compared to gymnosperms. Most notably, angiosperm height was more negatively influenced by increasing temperature variability, whereas gymnosperm height was negatively influenced by decreasing precipitation and increasing altitude. There was little evidence to suggest that shade tolerance influenced tree allometry except for very shade-intolerant trees which were taller for any given diameter. Tree allometry is plastic rather than fixed and scaling parameters vary around predicted central tendencies. This allometric variation provides insight into life-history strategies, phylogenetic history, and environmental limitations at biogeographical scales.

## Introduction

Size is perhaps the most fundamental trait of an organism (Niklas 1993). Height and stem diameter are components of tree size that are fundamental to processes ranging from individual performance to whole-ecosystem function. Tree height determines light capture (Moles et al. 2009), whereas stem diameter plays an important role in mechanical support (McMahon 1973; Niklas 1993) and water transport efficiency (Bullock 2000). The relationship between tree height and diameter is thought to reflect a trade-off between growth and survival (King et al. 2006). Trees that invest less in structural support can grow faster (Kobe 1999) and reach the canopy more quickly, yet insufficient structural support reduces the ability to resist buckling (Greenhill 1881).

The allometric scaling of tree height and stem diameter has been the subject of much theoretical and empirical debate (Henry and Aarssen 1999). Most hypotheses invoke (1) mechanical constraints which prevent tree buckling (Greenhill 1881; McMahon 1973), (2) hydraulic constraints that predict precipitation should influence tree architecture and allometry (Ryan et al. 2006), and (3) biophysical constraints arising from metabolic scaling theory which predict that tree height scales with diameter to the 2/3 power (Niklas and Spatz 2004). Yet, these predictions are difficult to reconcile with patterns of plant growth and competition (Enquist et al. 1998), and variation within and among tree species is not attributable solely to mechanical or hydraulic constraints (Niklas 1993; Niklas and Spatz 2004). Additional phenomena such as competition for light or water may alter tree allometry (Poorter et al. 2003). Thus, the variation of tree allometries warrants further exploration (Wang et al. 2006; Feldpausch et al. 2011; Kempes et al. 2011; Banin et al. 2012) especially as it relates to environmental conditions.

If trees optimize their growth strategies depending on the environment, then tree allometry is expected to vary predictably across environmental gradients (Banin et al. 2012). Indeed, recent studies have shown a dominant role of climate in determining variation in height–diameter allometry (e.g., Wang et al. 2006; Feldpausch et al. 2011; Banin et al. 2012). Specifically, temperature appeared to be a key driver of tree allometry in China with taller trees dominant in warmer climates (Wang et al. 2006), whereas temperature and precipitation seasonality increased the intercept but not the slope of height–diameter relationships across tropical forests world-wide (Feldpausch et al. 2011). However, the analysis of large-scale variation in tree allometry has only recently begun (Moles et al. 2009; Feldpausch et al. 2011; Banin et al. 2012; Lines et al. 2012), and it remains unclear whether large-scale variation in tree allometry is influenced by other factors such as phylogenetic or functional variation after accounting for environmental differences.

For example, gymnosperm and angiosperm trees are known to differ in their height–diameter allometry (King 1991) and in their sensitivity to competition (Bond 1989). Gymnosperms, specifically conifers, hold the world record for plant height and stem diameter (Niklas 1994) and, in general, gymnosperms have larger scaling exponents (Ducey 2012) and greater stem diameters at any given height (King 1991) compared to angiosperm trees. These allometric differences likely reflect differences in xylem anatomy, hydraulic safety margins, and drought and temperature tolerance (Hacke et al. 2005; Choat et al. 2012). It has even been hypothesized that the noticeably polarizing distributions of gymnosperm and angiosperm trees are a result of differences in allometric growth strategies (e.g., Bond 1989). Specifically, Bond (1989) suggested that gymnosperms should out-compete angiosperms in harsh environments characterized by freezing temperatures and extended droughts. In contrast, angiosperms should dominate over the inferior water transport of gymnosperms in warm, wet, light-limited environments. The angiosperm dominated eastern forests and western and high-elevation conifer forests of the United States seemingly support this hypothesis. However, few studies have provided broad comparisons across many species at biogeographical scales, and it remains to be seen if differences between angiosperm and gymnosperm allometry are primarily due to their differing evolutionary histories per se or environmental conditions.

Light demand is another known predictor of tree architecture (Poorter 2001; Poorter et al. 2003). Competition for light often results in vertical stratification within plant communities (Hara 1988; Aiba and Kohyama 1996). For example, shade-tolerant subcanopy species tend to be shorter for a given diameter as there is less competitive advantage in allocating biomass to height (King et al. 2006). In contrast, shade-intolerant trees grow taller, thinner, and faster (Poorter 2001; Poorter et al. 2003). Thus, in warm, wet, light-limited environments, it is expected that selection should lead to tree allometry mediated by shade tolerance. Arguably, these conditions should favor the broad, highly photosynthetic leaves of angiosperms. Although both shade tolerance and phylogeny are thought to influence tree architecture and allometry, few studies have assessed the joint effects of climate, relatedness, and shade tolerance on allometric variation.

Quantifying variation in tree allometry has both basic and applied value. First, quantifying allometric variation is important for understanding fundamental physiological trade-offs and for scaling individual to ecosystem-level processes (Givnish 1995). Second, understanding plant allometric variation is critical for improving regional and global estimates of forest biomass and carbon storage (Ducey 2012). To describe how tree allometries vary across biogeographical scales, we investigated the spatial distribution of scaling between height (H) and diameter (D) across the continental United States. Our specific objectives were to test (1) what allometric model provides the best fit, (2) whether abiotic differences (including temperature, precipitation, and seasonality) explain observed variation in H:D allometries across the United States or (3) whether floristic and functional groups can explain, in part, the differences in allometric scaling across biogeographical scales.

## Materials and Methods

**Figure 1:**
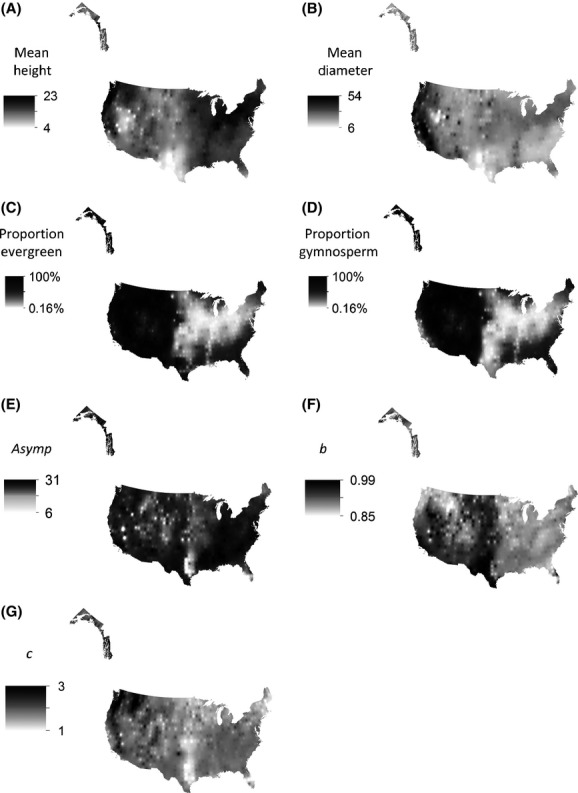
Inverse distance weighted interpolation maps of the United States representing mean tree height (A; m); mean tree diameter (B; cm); proportion of evergreen tree individuals (C; %); proportion of gymnosperms (D; %); and parameters from the fitted Gompertz equation: mean *asymptote* (E; maximum height, m); *b* (F; horizontal displacement of allometric curve); and *c* (G; allometric curve growth rate).

Individual georeferenced tree data were taken from the United States Department of Agriculture Forest Inventory and Analysis (FIA) Program, a national network of plots chosen to represent conditions across all forested lands of the United States (Woudenberg et al. 2010). This database includes a network of 212,272 plots encompassing 2,976,937 individual stems from 293 plant species. Plots cover all public and private forest land regardless of use (Bechtold and Scott 2005). We used the most recent available year for each plot in the database (mostly from years 2002 to 2007); thus, no individual tree is included more than once in this analysis. The diameter of each sample tree greater than 1 inch was measured at breast height (D), and the height (H) of trees was measured from the ground to the tip of the apical meristem using hypsometers, clinometers, or tape measurers for shorter trees (Woudenberg et al. 2010). Tree height was measured in feet and subsequently converted to meters; tree diameter was measured in inches and converted to centimeters. We excluded all nonliving trees and trees for which height was estimated from diameter or by eye. Species with <20 individuals in the entire dataset were excluded from the analyses as were 33 non-native species. Allometric models and scaling coefficients are highly sensitive to outliers (Niklas 2004); as a result, we also excluded strong outlying tree individuals based on Cook's distance and studentized residuals calculated using the standard deviation of tree diameter and tree height by species. These constraints eliminated 6.92% of individuals in the overall FIA dataset (remaining *n *=* *2,770,803 individual trees; Appendix 1). To visualize spatial patterns of tree height and diameter and scaling coefficients as well as the dominance of floristic and functional groups, interpolation maps were created in ArcGIS version 10.2 (ESRI 2014) using the inverse distance weighted interpolation method (Fig.1). More complex interpolation methods (e.g., kriging) produced similar results.

**Figure 2:**
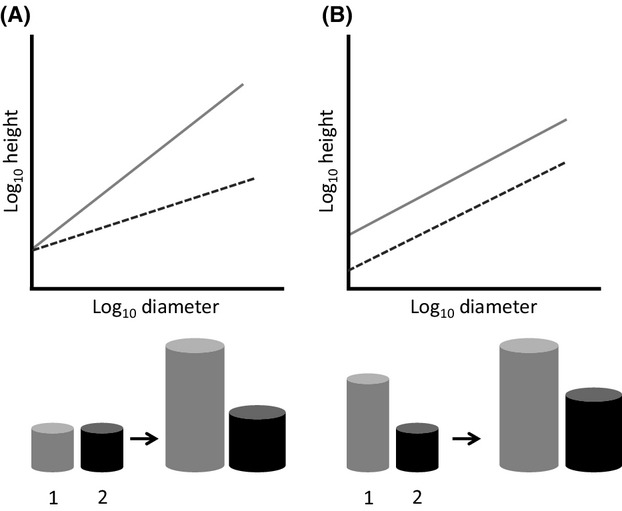
(A) At small diameters species 1 and 2 have similar H:D allometries. At larger diameters, however, species 1 and 2 diverge in their resource allocation strategies. (B) Species 1 and 2 have similar growth trajectories but different resource allocation strategies.

The basic scaling model for describing allometric relationships is the power function:

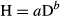
1(Huxley 1932) where H and D represent two traits of interest (here, tree Height and stem Diameter). On a log-log plot, *a* represents the intercept and *b* represents the scaling exponent or slope (Fig.2). Log-log transformations are commonly used to describe the relationship between H and D (Brown et al. 1989) and have been shown to provide better estimates of height relative to diameter compared to asymptotic functions for a subset of the FIA dataset (Purves et al. 2007) as well as for tropical trees around the world (Feldpausch et al. 2011). By fitting the power equation, we were also able to test whether the observed scaling coefficients were similar to those predicted from mechanical, hydraulic, and biophysical models. However, fitting the log-transformed power equation with ordinary least squares assumes that individual-tree residuals are log-normally distributed in the untransformed variable units (Russo et al. 2007). Adjusting the scaling coefficients to obtain unbiased values in the untransformed units has been proposed as has reduced major axis regression (see Ducey 2012). However, fitting the equation using reduced major axis regression is inappropriate as measurement errors in height (measured in feet, converted to meters) are likely much greater than those for diameter (measured in inches, converted to centimeters). In addition, several sources of variation should be accounted for in this basic model including variation due to location and species. Both of these sources of variation can be modeled as random effects, which may impact the scaling intercept, the scaling exponent, or both (Ducey 2012); thus, a least-squared approach is likely inappropriate.

To incorporate these considerations, we calculated height–diameter allometries using mixed-effects models fit by maximum likelihood for H onto D. The slope and intercept coefficients were allowed to vary by location, plot, or species which were included as random effects. This provided significantly better fit (likelihood ratio test, *P*-value < 0.001) compared to models where the error term was modeled with a constant variance. Likelihood ratio tests were used to compare scaling coefficients.

We also fit a three-parameter exponential function:

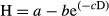
2where H is tree height, D represents stem diameter, *a* represents the asymptotic maximum tree height, *b* is the difference between the maximum and minimum tree height, and *c* is a curve fitting parameter. The fit of the power function is known to deviate for large stem sizes (Muller-Landau et al. 2006), and the three-parameter exponential function has been shown to outperform the power function for tropical trees (Banin et al. 2012). For comparison, we included three additional nonlinear mixed-effects models (Gompertz, Logistic, and Michaelis-Menten). Model fitting and selection followed an information-theoretic approach using Akaike's information criterion (AIC; Burnham and Anderson 2002).

After determining which allometric model provided the best fit, we were next interested in how climate, floristic, and functional groups determined allometric variation. As the exact physical location of FIA plots is ‘fuzzed and swapped’ due to privacy and security issues, each plot was assigned to one-degree grid cells based on its latitude–longitude coordinates. We selected a subset of noncorrelated climatic variables extracted from the 2.5-min resolution WorldClim dataset (Hijmans et al. 2005) and averaged within each one-degree grid cell. The final list included mean annual temperature (°C × 10), temperature seasonality (coefficient of variation of mean monthly temperatures), total annual precipitation (mm), precipitation seasonality (coefficient of variation of monthly precipitation totals), altitude (m), mean diurnal temperature range (BIO2; mean of monthly maximum–minimum temperatures, °C × 10), isothermality (BIO3; mean of monthly temperature range/mean annual temperature range °C × 100), and mean temperature of the wettest quarter (BIO8; °C × 10). To test for curvilinear responses to environmental variables, we included the square of each climatic variable as well. We also included floristic (angiosperm/gymnosperm) and functional groups (leaf habit and shade tolerance) as species-level covariates in the model. Shade tolerance classification was based on Niinements and Valladares (2006) and the U.S. Forest Service Silvics Manual (Burns and Honkala 1990) which relies heavily on Baker (1948). The shade tolerance classes were converted into an ordinal ranking (very tolerant* *=* *1, very intolerant* *=* *5). Most species were either deciduous angiosperms or evergreen conifers; thus, many of the comparisons unavoidably confound taxonomic effects with phenology. Coefficients were compared using likelihood ratio tests and AIC scores on reduced models.

To minimize correlations between scaling coefficients, diameter was centered, or normalized, to zero mean and unit variance and height was log-transformed. Both transformations achieved statistical assumptions of normality and homoscedacity. This also improved model convergence by standardizing the scale of measurement between height and diameter. All models were fit using maximum likelihood in order to compare models based on AIC scores (Zuur et al. 2009). Additionally, we quantified the coefficient of determination (*R*^2^) for each model (Lefcheck and Casallas 2014). We report both the marginal *R*^2^, which includes the variance of fixed factors, as well as the conditional *R*^2^, which includes the variance of both the fixed and random factors and, as a result, will always be higher (Nakagawa and Schielzeth 2013). To test whether climate, floristic, or functional groups were predictors of allometric relationships, factors were added in a stepwise fashion using linear mixed-effects models. For comparing models with the same fixed effects, factors were retained if (1) conditional *t*-tests and *F*-tests confirmed the significance of fixed effect terms, (2) a likelihood ratio statistic with the associated *P*-value indicated model improvement, and (3) the difference in the marginal *R*^2^ improved by more than 1%. Once the best model was determined, the model was refit using restricted maximum likelihood to provide more robust parameter estimates (Zuur et al. 2009). All statistical analyses were performed using the *nlme* (Pinheiro et al. 2013) and *domino* packages in R version 3.1.0 (R Core Team 2014) using Domino cloud computing (www.dominoup.com).

## Results

**Figure 3:**
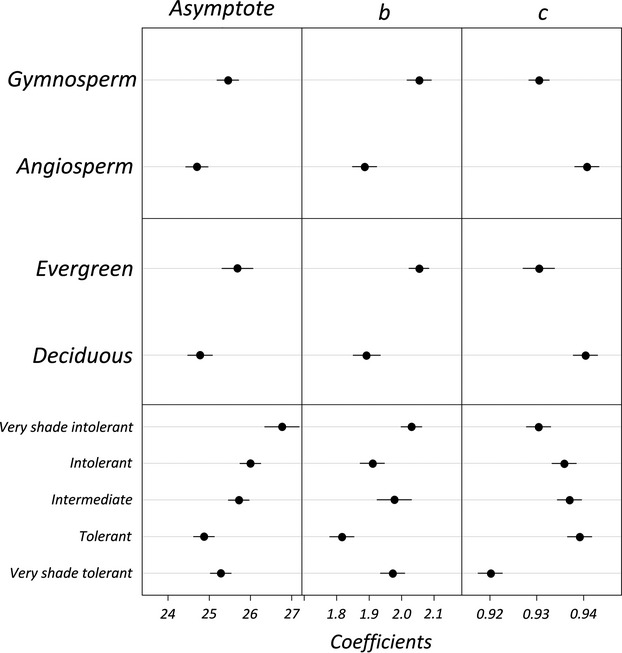
Mean and 95% confidence intervals for fixed effect coefficients for floristic and functional groups from nonlinear Gompertz mixed-effects models. Asymptote*, asymp,* represents maximum tree height, *b* represents the horizontal displacement of the allometric curve, and *c* represents the allometric curve growth rate. Floristic and functional groups were included in the model as fixed effects and plot as a random effect.

The Gompertz equation performed best followed by the three-parameter exponential equation (as evidenced by a lower AIC score; Appendix 2). Generally, asymptotic models out-performed the linear log-log power equation, however, the difference in AIC between the best fit and the worst fit was not great (Appendix 2). Maximum height (i.e., the Gompertz asymptote parameter, *a*) was highest for gymnosperms, evergreen, and very shade-intolerant species in paired likelihood ratio tests between floristic and functional groups (Fig.3, *P* < 0.01). This order was retained regardless of the model used (Appendix 2). Diameter at any given height (i.e., Gompertz parameter *b*) was highest for gymnosperm and evergreen species (Fig.3, *P* < 0.01). Curve growth rate (i.e., Gompertz parameter *c*) was slightly higher for gymnosperm (compared to angiosperm) and evergreen (compared to deciduous) species although this was modestly insignificant (*P* = 0.054 and *P* = 0.059, respectively). The observed scaling slope, *b*, of the power function ranged from 0.53 in angiosperms to 0.60 in gymnosperms; less than the 0.667 scaling slope predicted by the elastic-similarity and metabolic scaling theories and, for gymnosperms, higher than the scaling slope of 0.50 predicted by the stress-similarity model (Niklas 1993). In other words, only the observed scaling slope of the power function for angiosperms was similar to that predicted by the stress-similarity model (*P* = 0.0034).

Environment, gymnosperm versus angiosperm differences, and leaf habit (but not shade tolerance) also explained variation in tree allometry (Appendix 3). Climatic variables related to temperature and, to some extent, precipitation were correlated with allometric coefficients (Table1). Squared climatic terms were never retained in reduced models. In angiosperms, tree height was negatively affected by greater temperature seasonality and leaf habit (ANOVAs based on mean and standard deviation of effect coefficients; *P* < 0.01 for all comparisons). In gymnosperms, tree height was negatively affected by increasing altitude and decreasing mean precipitation. Shade tolerance was never retained in best-fit models explaining allometric variation (Table1).

**Table 1:** Summary of statistical tests using mixed-effects models to determine H:D allometric variation for log-transformed tree height (H, m) and mean centered diameter (D, cm) with random intercepts and slopes, plot as a random factor, and shade tolerance (not included in any of the best-fit models), angiosperm/gymnosperm (Clade), evergreen/deciduous (Phenology), and bioclimatic variables: altitude (m), mean temperature (°C × 10), mean precipitation (mm), precipitation seasonality, mean diurnal temperature range (Bio2), and isothermality (Bio3). For brevity, the best-fit model is reported for each functional or floristic group. AIC = Akaike's information criterion; *n* = number of tree individuals in each subsetted dataset

Model	Marginal *R*^2^	Conditional *R*^2^	AIC	Model structure	Fixed effects	Coefficient
All	0.55	0.75	−260,190	H ∽ D + Phenology + Altitude + SeasonPrecip + MeanTemp	Phenology	−0.06
*n* = 2,770,803					Altitude	−0.0001
					Season Precip	−0.0032
					Mean Temp	−0.0010
Gymnosperms	0.49	0.79	−348,174	H ∽ D + Altitude + Mean Precip	Altitude	−0.0001
*n* = 1,270,306					Mean Precip	0.0001
Angiosperm	0.60	0.77	−114,826	H ∽ D + Phenology + Bio3 + Bio2	Phenology	−0.22
*n* = 1,500,497					Bio3	−0.01
					Bio2	−0.0013
Evergreen	0.51	0.79	−320,509	H ∽ D + Clade + Altitude + Mean Precip	Clade	0.29
*n* = 1,292,397					Altitude	−0.0001
					Mean Precip	0.0001
Deciduous *n* = 1,478,406	0.60	0.76	−138,801	H ∽ D + Bio3	Bio3	−0.01

## Discussion

The geographic distribution of allometries can provide insight into the processes structuring plant communities (Givnish 1982; Kohyama 1993). While allometric studies are numerous, most are restricted to a few species at local or regional scales. As a result, interest in the biogeographical analysis of tree allometry has increased (e.g., Feldpausch et al. 2011; Banin et al. 2012). In this study, we determined the geographic distribution of plant growth strategies across the United States (Fig.1). We first asked what is the functional form of the allometric relationship between tree height and diameter? Although specific predictions for allometric scaling coefficients are derived using the power function (Brown et al. 2000), the power function may be inappropriate due to unaccounted variance of species or location. Asymptotic functions provided a better fit compared to the power function in this study and in temperate China (Wang et al. 2006). Similarly, asymptotic functions provided improved overall fit across tropical biomes (Banin et al. 2012), yet this was only confirmed in moist tropical forests, whereas in dry and wet tropical forests, the power function performed best (Feldpauch et al. 2011). These contrasting results may imply that environmental constraints determine whether allometry is logarithmic or asymptotic. To resolve this ongoing debate, more in-depth studies of the environmental conditions that lead to asymptotic or logarithmic allometry are needed. Regardless of the model used, it is clear that high plasticity and variation in allometric scaling is the rule rather than the exception (Niklas 1993; Brown et al. 2000).

Thus, determining the extent to which observed scaling parameters differ from predicted can help to elucidate underlying drivers of tree architecture and allometry. In this study, the lower than predicted allometric slopes suggest that limiting abiotic factors likely cause the observed reduction in optimal tree allometry (see Dudley and Gans 1991; McCarthy and Enquist 2007). Trees are known to optimize growth strategies and other life-history traits depending on environmental conditions. As a result, we expected to see predictable patterns of allometric variation across broad environmental gradients. Indeed, across the United States, mean tree height and diameter were lowest throughout parts of the arid West (Fig.1A,B). In arid environments, trees likely allocate more energy into belowground root structures at the cost of plant height (Schwinning and Ehleringer 2001). In other regions, reduced plant height (for a given diameter) was primarily explained by aridity and cold temperatures in Spain (Lines et al. 2012), by winter coldness in northeast China (Wang et al. 2006), and by aridity and seasonality within tropical regions (Feldpausch et al. 2011; Banin et al. 2012). Together, these results indicate a convergence of growth strategies in unfavorable environments (Grime 2002) and suggest that abiotic factors largely influence tree architecture and allometry at large spatial scales.

While the present study provides evidence that abiotic factors are a primary driver of allometric variation, our results and others (e.g., Gómez-Aparicio et al. 2011; Lines et al. 2012; Coll et al. 2013) also suggest that the degree to which abiotic factors influence tree allometry depends largely on the contrast between angiosperms and gymnosperms and their divergent stem anatomy. The complex wood anatomy of angiosperms contains large vessels that are less resistant to solute flow and freezing-induced embolization compared to gymnosperm tracheids (Wang et al. 1992) which reduce transport efficiency and relative growth rate of gymnosperms. Bond (1989) suggested that gymnosperms should out-compete angiosperms in harsh environments while angiosperms should dominate over the inferior water transport of gymnosperms in warmer, wetter climates. Gymnosperms experienced a reduction in tree height at higher altitudes while angiosperms experienced a reduction in tree height in more thermally variable climates. Together, these findings point to underlying differences that may help to explain why angiosperms and gymnosperms have such polarized geographical distributions. Caution in extrapolating these findings to explain geographical distribution is needed unless a more direct mechanism can be linked to differences in tree allometry between gymnosperms and angiosperms such as, for example, differences in hydraulic conductivity, wood density, and cavitation (e.g., Swenson and Enquist 2007; Swenson and Weiser 2010; Choat et al. 2012; but see Becker 2000). Also, gymnosperm and angiosperm species co-occur. In these environments, the taller height of gymnosperm trees at smaller diameters (as evidenced by a greater Gompertz parameter *b*) may help to offset the disadvantage of slow growth and increased light competition when co-occurring with faster growing angiosperms. Further, the differing allometric growth strategies in angiosperms and gymnosperms may influence responses to global warming. Recent large-scale studies have reported contrasting responses of Mediterranean tree growth to temperature in angiosperm and coniferous trees (Gómez-Aparicio et al. 2011; Coll et al. 2013). In this study, we show that angiosperm height was more sensitive to increases in temperature variability (negatively), whereas gymnosperm height was more sensitive (negatively) to decreases in mean precipitation. Thus, the predicted decrease in precipitation and increase in temperature across large portions of the United States may result in reduced performance of both broad-leaved angiosperms and gymnosperms due to complex climatic responses. Although studies from Mediterranean plant communities also predicted reduced performance in conifers with a decrease in precipitation (Gómez-Aparicio et al. 2011; Coll et al. 2013), generalizations remain difficult due to the limited number of biogeographical comparisons between angiosperm and gymnosperm allometry.

Contrary to our expectations, we found little evidence to suggest that tree allometry varies dramatically due to differences in shade tolerance, with the exception of very shade-intolerant trees which had higher maximum heights and a higher allometric growth curve (i.e., Gompertz parameter *c*) indicating a strategy that maximizes vertical growth. Shade tolerance was also shown to have little influence on tree allometry for northeastern United States tree species (Ducey 2012). This is difficult to reconcile with previous studies that emphasize light demand as a key trait determining community assembly (e.g., Aiba and Kohyama 1996). It is possible that variation in allometric strategies due to shade tolerance is either confounded by (1) the effects of phenology and phylogeny, (2) the effects of climate largely predominate over the effects of shade tolerance at the scales measured here, or (3) the challenges of classifying functional types, such as shade tolerance, masks observed plant plasticity and ontogenetic variation. An obvious challenge is to explain the ecological and physiological mechanisms involved (e.g., Poorter et al. 2003; Niinements and Valladares 2006; Osunkoya et al. 2007).

In short, we quantified large-scale patterns of tree growth strategies and provided a test of the underlying drivers of tree allometry across a broad geographical scale. We showed that climatic gradients interact with plant phylogeny and function to modulate height–diameter relationships. In general, altitude, mean annual precipitation, and temperature seasonality were predominant factors influencing tree allometry. The magnitude and importance of climate, however, varied most strongly between gymnosperms and angiosperms. Understanding climatic modulation of tree allometry is required for developing regional biomass and carbon equations and for predicting how plant communities will respond to changing environments. Biogeographical studies of tree allometry, however, have several inherent limitations including the limited availability of local microclimatic conditions that likely influence tree allometry especially as it relates to competition for light. The increasing availability of global databases and large networks of vegetation monitoring should overcome some of these challenges. A necessary next step is to determine the fundamental physiological mechanisms responsible for allometric differences between phylogenetic and functional groups across broad climatic gradients.

## Figures and Tables

**Figure:**
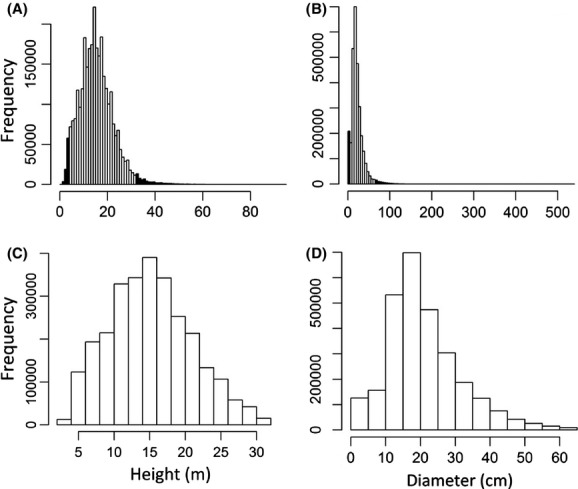
Frequency distributions of tree height (m) and diameter (cm) in the FIA dataset before subsetting (A and B) and after (C and D). Black shaded bars represent the data removed in subsequent analyses, representing 6.92% of the total dataset.

## Appendix 2

**Table:** Comparison of nonlinear mixed effects models for five functional forms. Floristic (angiosperm/gymnosperm) and functional (evergreen/deciduous) groups were included as fixed effects and plot as a random effect. The standard deviation (SD) of each random term (*a*, *b*, and, where relevant, *c*) and residuals is given. AIC = Akaike's information criterion; *n* = number of tree individuals in each subsetted dataset

				Fixed effects											
				Angiosperm *n* = 1,500,497			Gymnosperm *n* = 1,270,306			Evergreen *n* = 1,292,397			Deciduous *n* = 1,478,406		
Eq.	AIC	Random	SD	a	b	c	a	b	c	a	b	c	a	b	c
*H* = *a*D^*b*^	15,250,159	a	0.42	2.37	0.53	NA	3.21	0.60	NA	3.25	0.60	NA	2.35	0.53	NA
		b	0.03												
		Residual	3.79												
MicMen	15,218,854	a	2.53	36.82	25.02	NA	41.88	37.6	NA	41.1	37.13	NA	37.16	24.96	NA
		b	6.29												
		Residual	3.77												
3-par exp	15,197,342	a	0.31	28.32	0.65	−3.24	28.95	2.03	−3.39	28.73	2.01	−3.4	28.51	0.82	−3.24
		b	0.69												
		c	0.45												
		Residual	3.76												
Gompertz	15,185,010	a	0.30	25.13	1.88	0.94	25.26	2.06	0.93	25.46	2.04	0.93	24.84	1.88	0.94
		b	0.09												
		c	0.003												
		Residual	3.75												
Logistic	15,192,597	a	0.48	23.97	12.98	9.31	23.97	15.63	10.51	24.24	15.46	10.38	25.53	12.98	9.33
		b	1.30												
		c	0.60												
		Residual	3.75												

## Appendix 3

**Table:** Model fitting selection of H:D allometry. Model fitting using mixed effects models to examine variation between log-transformed height and mean centered diameter. Model fitting follows Feldpausch et al. (2011) for comparison with tropical tree architecture. Models M10-M40 include plot as a random effect. Best-fit models meeting selection criteria for each subgroup are indicated in bold. Analogous model fitting was performed on the subsetted data based on clade (gymnosperm/angiosperm), leaf habit (evergreen/deciduous), and shade tolerance. On subsetted datasets, redundant fixed effects were thus removed from the model (e.g., for the angiosperm subset, “clade” was not included as a predictor variable)

Model	Marginal *R*^2^	Conditional *R*^2^	AIC	Model structure
1. Is their hierarchical structure to the data?				
M1	0.341	0.440	3,156,999	H ∽ 1
**M2**	**0.480**	**0.510**	**1,253,216**	**H ˜ D**
M3	0.278	0.407	549,213	H ∽ D, random = ∽1|D
2. Does H:D allometry differ by location, species, clade, leaf habit, shade tolerance, plot?				
M4	0.359	0.632	672,123	H ∽ D, random = lat-lon
M5	0.413	0.660	14,590,705	H ∽ D, random = species
M6	0.489	0.506	1,200,087	H ∽ D, random = clade
M7	0.488	0.515	1,156,490	H ∽ D, random = leaf habit
M8	0.483	0.504	1,218,054	H ∽ D, random = shade tolerance
**M9**	**0.491**	**0.752**	−**208,065**	**H ˜ D, random = plot**
3. Does H:D allometry differ by floristic and functional groups?				
M10	0.483	0.760	−273,169	H ∽ D + shade tolerance
M11	0.498	0.750	−212,773	H ∽ D + clade
**M12**	**0.508**	**0.750**	−**229,391**	**H ˜ D + leaf habit**
M13	0.498	0.757	−288,762	H ∽ D + shade tolerance + clade + leaf habit
M14	0.500	0.758	−301,803	H ∽ D + shade tolerance + leaf habit
4. Does H:D allometry differ by floristic and functional groups and climate?				
**M15**	**0.531**	**0.751**	−**247,824**	**H ˜ D** + **leaf habit** + **Altitude**
M16	0.508	0.750	−236,527	H ∽ D + leaf habit + Mean Temp
M17	0.520	0.752	−239,967	H ∽ D + leaf habit + Season Temp
M18	0.516	0.750	−241,403	H ∽ D + leaf habit + Mean Precip
M19	0.528	0.749	−248,625	H ∽ D + leaf habit + Season Precip
M20	0.525	0.752	−242,447	H ∽ D + leaf habit + Bio2
M21	0.526	0.753	−241,931	H ∽ D + leaf habit + Bio3
M22	0.514	0.750	−239,178	H ∽ D + leaf habit + Bio8
4a. Are there non-linear climatic responses?				
M15a	0.531	0.751	−247,824	H ∽ D + leaf habit + Altitude + Altitude^2^
M16a	0.508	0.750	−236,527	H ∽ D + leaf habit + Mean Temp + Mean Temp^2^
M17a	0.520	0.752	−239,967	H ∽ D + leaf habit + Season Temp + Season Temp^2^
M18a	0.516	0.750	−241,403	H ∽ D + leaf habit + Mean Precip + Mean Precip^2^
M19a	0.528	0.749	−248,625	H ∽ D + leaf habit + Season Precip + Season Precip^2^
M20a	0.525	0.752	−242,447	H ∽ D + leaf habit + Bio2 + Bio2^2
M21a	0.526	0.753	−241,931	H ∽ D + leaf habit + Bio3 + Bio3^2
M22a	0.514	0.750	−239,178	H ∽ D + leaf habit + Bio8 + Bio8^2
4b. Step-wise forward selection of climatic terms				
M23	0.540	0.752	−250,580	H ∽ D + leaf habit + Altitude + Mean Temp
M24	0.539	0.751	−250,503	H ∽ D + leaf habit + Altitude + Season Temp
M25	0.532	0.750	−248,403	H ∽ D + leaf habit + Altitude + Mean Precip
**M26**	**0.541**	**0.749**	−**255,745**	**H ˜ D** + **leaf habit** + **Altitude** + **Season Precip**
M27	0.534	0.751	−248,545	H ∽ D + leaf habit + Altitude + Bio2
M28	0.539	0.752	−250,354	H ∽ D + leaf habit + Altitude + Bio3
M29	0.531	0.751	−247,835	H ∽ D + leaf habit + Altitude + Bio8
**M30**	**0.554**	**0.751**	**−260,190**	**H ˜ D** + **leaf habit** + **Altitude** + **Season Precip** + **Mean Temp**
M31	0.550	0.750	−258,639	H ∽ D + leaf habit + Altitude + Season Precip + Season Temp
M32	0.541	0.749	−255,833	H ∽ D + leaf habit + Altitude + Season Precip + Mean Precip
M33	0.544	0.749	−256,343	H ∽ D + leaf habit + Altitude + Season Precip + Bio2
M34	0.548	0.750	−257,743	H ∽ D + leaf habit + Altitude + Season Precip + Bio3
M35	0.541	0.749	−255,744	H ∽ D + leaf habit + Altitude + Season Precip + Bio8
M36	0.554	0.751	−260,244	H ∽ D + leaf habit + Altitude + Season Precip + Mean Temp + Season Temp
M37	0.555	0.751	−261,240	H ∽ D + leaf habit + Altitude + Season Precip + Mean Temp + Mean Precip
M38	0.554	0.751	−260,291	H ∽ D + leaf habit + Altitude + Season Precip + Mean Temp + Bio2
M39	0.554	0.750	−260,745	H ∽ D + leaf habit + Altitude + Season Precip + Mean Temp + Bio3
M40	0.554	0.751	−260,376	H ∽ D + leaf habit + Altitude + Season Precip + Mean Temp + Bio8
